# Ergonomics of Various Modalities for Ear Surgery

**DOI:** 10.1002/oto2.162

**Published:** 2024-07-04

**Authors:** Matthew E. Lin, Sheng Zhou, Seiji Kakeheta, Tsukasa Ito, Seiji B. Shibata

**Affiliations:** ^1^ Department of Head and Neck Surgery David Geffen School of Medicine at University of California Los Angeles Los Angeles California USA; ^2^ Caruso Department of Otolaryngology–Head and Neck Surgery Keck School of Medicine of the University of Southern California Los Angeles California USA; ^3^ Department of Otorhinolaryngology Ota General Hospital Kawasaki Kanagawa Japan; ^4^ Department of Otolaryngology, Head and Neck Surgery Yamagata University Faculty of Medicine Yamagata Japan

**Keywords:** endoscope, ergonomic, exoscope, microscope, middle ear surgery

## Abstract

**Objective:**

Evaluate ergonomic differences of various modalities for performing middle ear surgery.

**Study Design:**

Observational study.

**Setting:**

Two academic tertiary care centers.

**Methods:**

Attending physicians and residents performing middle ear surgery were photographed intraoperatively. Intraoperative photographs were analyzed using the validated Rapid Upper Limb Assessment (RULA) tool to measure musculoskeletal disease (MSD) risk. Descriptive statistics and significance testing were used to characterize and compare ergonomic differences between surgical modalities. Multivariable ordinal regression was performed to assess factors associated with increased MSD risk, as determined by the final RULA score.

**Results:**

Most of our 110 intraoperative photos featured attendings (82.7%) performing combined middle ear surgery and mastoidectomy (60.0%). Body angles and the final RULA score varied significantly among modalities. On subset analysis, microscopic surgery exhibited significantly worse wrist, trunk, and neck angles compared to endoscopic and exoscopic surgery. Exoscopic surgery had significantly lower final RULA scores than both endoscopic and microscopic surgery, indicating significantly lower MSD risk. Microscopic and endoscopic surgery final scores did not vary significantly. In a multivariable ordinal regression of factors associated with increased RULA score, exoscopic surgery had statistically significantly less ergonomic risk relative to microscopic surgery (odds ratio = 0.12, 95% confidence interval = [0.03‐0.43]).

**Conclusion:**

Exoscopic, endoscopic, and microscopic surgery all featured low ergonomic risk, although exoscopic middle ear surgery demonstrated the lowest risk profile among studied surgical modalities. This demonstrates the importance of using each modality in combination with other ergonomic interventions to provide meaningful musculoskeletal benefits.

Ergonomics is a field defined by its focus on reducing occupational risk of injury to improve quality of life. What started first as an emphasis on ergonomics in general surgery has become significantly more important in otolaryngology. In a 2018 study of 137 Canadian otolaryngologists, 97% reported experiencing physical pain.^1^ Similarly, a 2017 study of 141 otolaryngology residents found that 81% endorsed noting significant cervical neck symptoms.^2^

Otologic surgery includes several unique ergonomic challenges. The narrow working space often demands awkward positioning of wrists and arms. Furthermore, otologic surgeons often drill an action shown to decrease blood volume to the operator's extremity through vasoconstriction. Additionally, otologists perform repetitive motions throughout surgeries, whether when using curettes or elevating flaps. Altogether, these factors magnify otologists' ergonomic risk.^3^, ^4^

These considerations accentuate the importance of investigating surgical modalities to improve the ergonomics of otologic surgery. Traditionally, otologic surgery was performed with operating microscopes. More recently, the endoscope has been adapted for use in the operating room. Arrighi‐Allisan et al found resident necks and backs were more flexed when using the microscope compared to the endoscope.^5^ However, the exoscope represents an even newer modality now used in Europe and Asia. Exoscopes allow for the use of 3‐dimensional (3D) cameras to magnify the surgical field and project it onto a video tower. Surgeons use 3D polarization glasses to operate off the video tower.^6^, ^7^ This modality has not been ergonomically compared to others in the literature. As such, we sought to characterize the neck, arm, and trunk angles for these 3 modalities and determine the total ergonomic risk score for each using the validated Rapid Upper Limb Assessment (RULA). Although a variety of ergonomic assessments exist, we elected to use the RULA due to its comprehensive evaluation, intuitive interpretation, and prevalence of use in previous ergonomic literature.^5^, ^8^

## Methods

This was an international observational study of otolaryngology attending and resident ergonomic positioning during middle ear surgery at 2 different tertiary care centers in Japan and the United States. This study received approval by the Institutional Review Board (HS‐23‐00047).

**Figure 1:**
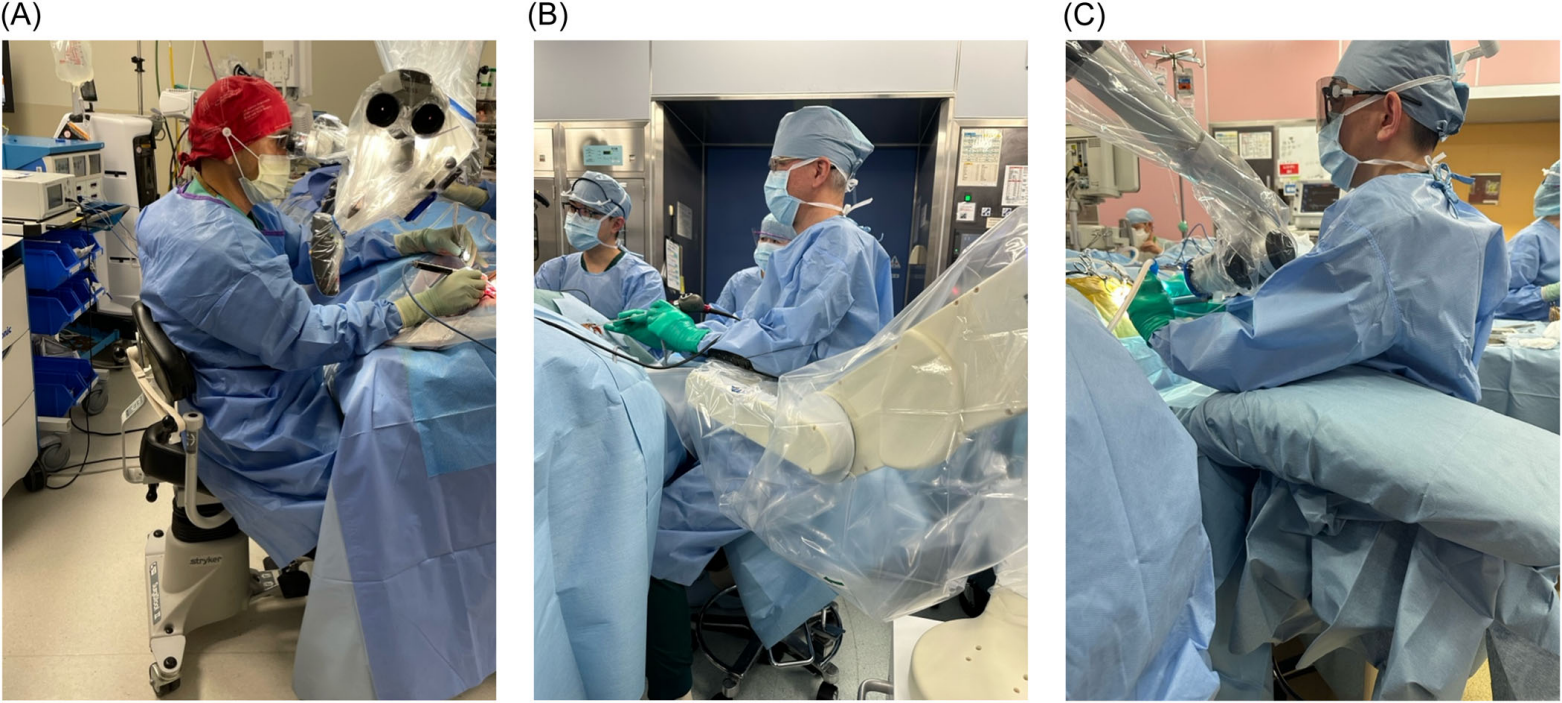
Examples of intraoperative photos. (A) Microscopic surgery. (B) Endoscopic surgery. (C) Exoscopic surgery.

Eight fellowship‐trained otology‐attending physicians and 3 otolaryngology residents at 2 academic medical centers were included in this study. Prior to surgery, physicians were asked if 1 of 2 study members (S.Z. and M.E.L.) could record surgeon positioning during the exoscopic, endoscopic, and/or microscopic portions of middle ear surgery. The exoscope used was the Olympus ORBEYE 4K. Neither S.Z. or M.E.L. had any supervisory role. Ergonomic positioning was collected by photographing surgeon positioning during the relevant portions of these cases—when an exoscope, endoscope, or microscope was used—at regular 15‐minute intervals using a password‐protected mobile phone with a high‐resolution camera. Photos were taken parallel to the sagittal plane of the operating surgeon such that all extremities on the surgeon's documented side, as well as his or her trunk, hips, head, and neck, were visible. Multiple pictures were taken from different points, but in the same parallel plane, when all critical points were unable to be simultaneously visualized. Significant care was taken to ensure no identifying patient information was shown in any photograph. Example photos depicting study authors are shown in Figure 1.

Each photograph was assigned a unique 4‐digit ID as determined by the default file name. Surgeon experience (year as attending or resident), surgical modality (exoscope, endoscope, or microscope), and type of surgery were separately collected for each photograph. Exoscope and endoscope data was collected at the University of Yamagata, whereas microscope and endoscope data was collected at the University of Southern California.

Intraoperative photographs were analyzed by S.Z. and M.E.L. in ImageJ (National Institutes of Health) using the RULA Tool, an objective and validated tool for measuring body posturing and musculoskeletal disease (MSD) risk.^9^, ^10^ This tool did not require any training in ergonomic analysis. It also minimizes the involvement of the operating surgeon as it does not require any wearable material. The RULA tool consists of 17 separate measurements which are calculated into a series of 7 subsite scores (arm, elbow, wrist, wrist/arm combined, neck, trunk, neck/trunk combined) used to produce a single final overall score of overall MSD risk. Higher overall scores corresponded to worse body positioning. Final scores also corresponded to the level of MSD risk (1‐2 = negligible risk with acceptable posture; 3‐4 = low risk with further investigation as change may be needed; 5‐6 = moderate with risk further investigation and change soon; 7 = high risk and need to investigate and implement change). Subsite angle measurements were also measured as part of the RULA score assessment but were also separately collected for analysis.

Descriptive statistics were used to characterize ergonomic scores stratified by surgical modality, level of training, and RULA subsite. Analysis of variance was utilized for parametric data (arm and elbow angles), whereas Kruskal‐Wallis was used among nonparametric data (wrist, neck, and trunk angles; final RULA score) to assess ergonomic differences between all 3 surgical modalities. Similarly, unpaired *t‐tests* were used for parametric data, and the Wilcoxon rank‐sum test with continuity correction was utilized for nonparametric data to isolate differences between modalities to determine which modality is most ergonomic. Multivariable ordinal regression of factors associated with increased MSD risk, as determined by the final RULA score, was performed to control for surgeon level (trainee vs attending) and surgical modality (microscope, endoscope, and exoscope). Statistical analysis was performed in STATA (StataCorp LLC), R (R Foundation), and Microsoft Excel (Microsoft).

## Results

This cross‐sectional assessment included 22 middle ear surgeries performed by 11 surgeons (Table 1). Scopes were used intraoperatively for an average of 78.00 minutes (SD = 37.28); exoscopes were used in 27.27% of cases. Surgeons were primarily male attending physicians (72.73%) with an average of 13.64 years (SD = 10.44) of surgical experience.

**Table 1:** Participant and Surgical Characteristics

Characteristic	n (%)
Number of surgeries	22
Modality	
Microscope	10 (45.45)
Endoscope	6 (27.27)
Exoscope	6 (27.27)
Surgery	
Stapedectomy	1 (4.55)
Canalplasty	1 (4.55)
Tympanoplasty	5 (22.73)
Tympanomastoidectomy	6 (27.27)
Cochlear implant	9 (40.91)
Duration of scope use, min (mean, SD)	78 (37.28)
Number of surgeons	11
Sex	
Male	8 (72.73)
Female	3 (27.27)
Country of practice	
United States	4 (36.36)
Japan	7 (63.64)
Surgical experience	
Attending	8 (72.73)
Resident	3 (27.27)
Surgical experience, y (mean, SD)	13.64 (10.44)

Characteristics of our 110 photos of intraoperative surgeon ergonomic positions are detailed in Table 2. Microscopic surgery was most documented (n = 52, 47.27%), and attendings were featured in a vast majority of our evaluations (n = 91, 82.73%). Most photos documented both middle surgery and mastoidectomy (n = 66, 60.00%), with fewer being exclusively middle ear surgery (n = 44, 40.00%).

**Table 2:** Characteristics of Ergonomic Evaluation Pictures (n = 110)

Characteristic	n (%)
Surgical modality	
Microscope	52 (47.27)
Endoscope	28 (25.45)
Exoscope	30 (27.27)
Surgeon experience	
Attending	91 (82.73)
Resident	19 (17.27)
Surgery	
Middle ear surgery with mastoidectomy	81 (73.64)
Middle ear surgery only	29 (26.36)

**Figure 2:**
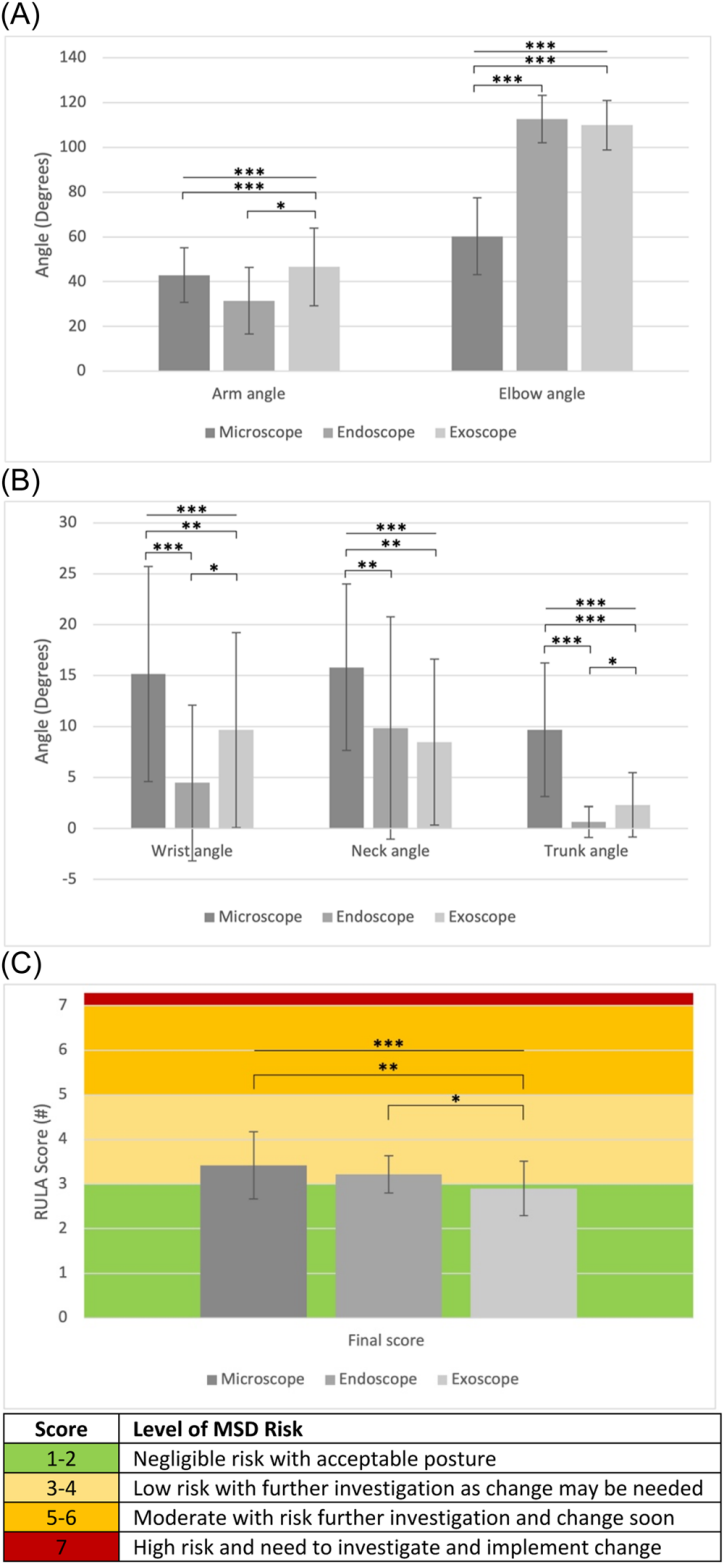
Average surgeon bodily angles and final RULA score stratified by surgical modalities (microscope, endoscope, exoscope). (A) Arm and elbow. (B) Wrist, neck, and trunk. (C) Final score. Score interpretation and clinical implications are depicted. MSD, musculoskeletal disease; RULA, Rapid Upper Limb Assessment. *p < .05, **p < .01, and ***p < .001.

Figure 2 shows average surgeon body angles stratified by all 3 surgical modalities; significant differences (*P* < .001) existed in average arm, elbow, wrist, neck, and trunk angles. Significant differences were also exhibited in the average RULA score between modalities (*P* < .001). Higher scores correspond to a higher MSD risk. The most ideal arm angle is 20° or less from the trunk. Endoscopic surgery featured an average arm angle (31.50, SD = 14.86) significantly lower than that of microscopes (mean = 42.94, SD = 12.20, *P* < .001) and exoscopes (mean = 46.60, SD = 17.35, *P* = .027). Microscopes featured the lowest and most ergonomic average elbow angle (60.31, SD = 17.18), which was significantly lower than that of endoscopes (mean = 112.71, SD = 10.65, *P* < .001) and exoscopes (mean = 109.93, SD = 11.11, *P* < .001).

Microscopic surgery also featured the greatest average wrist angle (15.15, SD = 10.58) and neck angle (15.80, SD = 8.18). The ideal wrist angle is 0° in a horizontal neutral position. The ideal neck angle is 0° to 10° from the trunk. The wrist and neck angles for microscopic surgery were both significantly higher than that of endoscopic surgery (wrist: mean = 4.46, SD = 7.62, *P* < .001; neck: mean = 9.86, SD = 10.90, *P* = .001) and exoscopic surgery (wrist: mean = 9.67, SD = 9.56, *P* < .022; neck: mean =8.47, SD = 8.14, *P* = .001). Similarly, microscopic surgery featured the worst trunk angle (mean = 9.69, SD = 6.56), which was significantly higher than that of endoscopic surgery (mean = 0.64, SD = 1.50, *P* < .001) and exoscopic surgery (mean = 2.30, SD = 3.14, *P* < .001). While endoscopic surgery featured significantly better average wrist angle (*P* = .026) and trunk angle (*P* = .014) relative to that of exoscopic surgery, there were no significant differences in neck angle between the 2 modalities. Finally, exoscopic surgery had the lowest final RULA score (mean = 2.90, SD = 0.61)—indicative of negligible to low ergonomic risk with acceptable posture. This was significantly lower than that of endoscopic surgery (mean = 3.22, SD = 0.42, *P* = .046) and microscopic surgery (mean = 3.42, SD = 0.75, *P* = .005), whose scores were indicative of low ergonomic risk with the need for further investigation and potential change. The average final RULA score for microscopic and endoscopic surgery was not significantly different.

A multivariable ordinal regression of factors associated with increased RULA score, controlling for surgical modality and surgeon experience, is documented in Table 3. Relative to microscopic surgery, exoscopic surgery was associated with a significantly lower likelihood of increased RULA score, indicating a significant reduction in MSD risk (odds ratio [OR] = 0.12, 95% confidence interval [CI] = [0.03‐0.43], *P* = .001). Mastoidectomy was also associated with significantly lower MSD risk (OR = 0.27, 95% CI = [0.09‐0.84], *P* = .024). Endoscopic surgery was not associated with significantly different MSD risk relative to microscopic surgery (OR = 0.62, 95% CI = [0.23‐1.67], *P* = .343], nor was resident‐level surgical skill relative to attending physician surgical skill (OR = 0.77, 95% CI = [0.26‐2.33], *P* = .646).

**Table 3:** Multivariable Ordinal Regression of Factors Associated With Increased Rula Score

Characteristic	Odds ratio	95% CI	*P* value
Surgical modality			
Microscope	Reference	Reference	Reference
Endoscope	0.44	[0.16‐1.25]	.125
Exoscope	0.14	[0.04‐0.51]	**.003**
Mastoidectomy			
No	Reference	Reference	Reference
Yes	0.27	[0.09‐0.84]	**.024**
Surgeon experience			
Attending physician	Reference	Reference	Reference
Resident physician	1.04	[−1.11 to 1.19]	.950

*Note*: Bold values are statistically significant.

Abbreviation: CI, confidence interval.

## Discussion

In this study, we assessed the ergonomic differences between microscopic, endoscopic, and exoscopic middle ear surgery using the validated RULA tool. Average arm, elbow, wrist, and neck angles significantly differed across the 3 surgical modalities studied. Two‐modality subset analysis also demonstrated many significant differences, with the fewest reported when comparing endoscopic and exoscopic surgery. The final RULA score, where higher scores suggest increased ergonomic risk, also significantly varied between modalities. The final RULA score for exoscopic surgery indicated negligible to low ergonomic risk with acceptable posture. This contrasted with endoscopic and microscopic surgery, whose scores suggested low ergonomic risk with the recommended investigation and potential positional change. There were no significant differences between microscopic and endoscopic surgery. On a multivariable ordinal regression assessing factors associated with increased RULA score, exoscopic surgery but not endoscopic was significantly less associated with increased score relative to microscopic surgery.

Previous studies have used the validated RULA tool to assess aspects of ergonomic differences between microscopic and endoscopic surgery.^4^, ^5^ One study by Arrighi‐Allisan et al found residents experienced significantly more flexed necks and backs during microscopic surgery when compared to endoscopic surgery; although no significant differences in angle existed between attendings, they did report significantly higher postoperative pain after using the microscope.^5^ Although we did not stratify our angle, position, and final RULA score data by level of experience on significance testing, our multivariable model suggests level of training was not significantly associated with worse ergonomic positioning, a finding more in line with findings from the general surgery literature.^11^ This finding may stem from the relative novelty of surgical ergonomic considerations within otolaryngology and other specialties, especially considering the high rates of poor posturing and ergonomics found among otolaryngologists.^3^, ^12^, ^13^ However, interventions such as resident ergonomic teaching programs and intraoperative breaks have shown promise in improving intraoperative ergonomic positioning, and if habitually implemented among trainees, may result in improved musculoskeletal outcomes among trainee otolaryngologists.^14^, ^15^

Another study by Joo et al found no significant differences in neck flexion and extension angles or time spent in high‐risk neck positions between endoscopic and microscopic surgery.^16^ This study found significant differences in all extremity and trunk angles between microscopic and endoscopic surgery. Specifically, microscopic surgery reported significantly higher angles in all categories compared to endoscopic surgery except for in elbow score. The differences between these surgical modalities in our results may be due to the “heads‐up” nature of endoscopic surgery as compared to the “heads‐down” nature of microscopic surgery: whereas surgeons can view the endoscope monitor in a relatively neutral position around eye level, the often must lean forward and downwards when using operative microscopes.^15^ As such, endoscopic surgery inherently offers an opportunity for smaller and more ergonomic intraoperative neck and back angles. Furthermore, the reduced size and increased maneuverability endoscopes provide relative to operative microscopes, as well as the ability to customize operating room setup during endoscopic middle ear surgery, may allow surgeons to better optimize their bodily positioning during surgery.^17^ Thus, as microscopic surgery has long been associated with poor ergonomics, endoscopic surgery may provide some ergonomic benefits without compromising clinical outcomes.^3^, ^15^, ^17^

These proposed benefits, however, did not translate into reduced ergonomic risk in our multivariate model. This result may be due to the additional variables and considerations included in the validated final RULA score, which represented a composite of individual body part subscores.^9^, ^10^ First, the validated scoring system scores body angles based on specific ranges such that significant differences in measured position intraoperatively may not necessarily correspond to significantly different subscores. These subscores are also adjusted based on criteria such as other positional, muscle use, and load‐carrying factors not elucidated when only considering body angles. Finally, the final RULA score is calculated from individual subscores based on a rubric that further weighs the impact of each subscore on the overall final score. Whereas operating microscopes are mounted on floor stands and can be suspended without considerable surgeon effort, endoscopes wholly rely on operating surgeons to hold and position. As such, the need for surgeons to constantly balance the endoscope's weight intraoperatively for extended periods of time increases ergonomic strain and adds additional ergonomic risk absent in microscopic surgery.^18^ Whereas previous studies have focused on specific key body angles when assessing musculoskeletal disease risk, doing so without considering a plethora of other factors may paint an incomplete and less nuanced picture of bodily positioning during middle ear surgery.^5^, ^16^ As such, although endoscopic surgery may result in certain improvements in body angle and positioning relative to microscopic surgery, its ergonomic benefits may be incomprehensive and suggest a continued need for ergonomic innovation within otology.^19^

Our multivariable model also demonstrated that exoscopic surgery is associated with a significantly lower likelihood of MSD relative to microscopic surgery, a finding corroborated by the significantly better body positioning found when directly comparing the 2 surgical modalities through significance testing. Like endoscopes, exoscopes also offer a heads‐up operative view that decreases neck and back strain relative to heads‐down operative microscopes.^20^, ^21^ However, exoscopes provide the additional benefit of not requiring to be held intraoperatively and provide a larger working space, whereas endoscopes may require 1‐handed surgery when surgical assistants are unavailable. By taking up less room compared to a microscope, exoscopes allow for more ideal surgeon arm and elbow positioning intraoperatively. The exoscope's ergonomic benefits may also extend to surgeon assistants, who may be able to adopt a more relaxed position intraoperatively when the exoscope is used relative to the operative microscope.^22^ The exoscope has been described to have ergonomic superiority, increased physician comfort, improved teaching ability, and comparable clinical outcome as compared to the microscope in the neuro‐otologic, otolaryngologic, and neurosurgical literature.^20^, ^21^, ^22^, ^23^, ^24^, ^25^, ^26^, ^27^ These benefits, however, were primarily assessed through subjective physician experience and described based on the intraoperative setup of both technologies. We corroborate and build upon these previous studies by using the comprehensive and validated RULA tool to produce similar findings. Although endoscopic and exoscopic surgery performed similarly when assessing individual body angles and positions, exoscopic surgery exhibited a statistically significantly lower overall RULA score.^9^, ^10^, ^20^, ^21^

Despite these promising findings for exoscopes, the small differences in average RULA scores between exoscopes and microscopes suggest the ergonomic benefits exoscopic surgery provides may be smaller than previously suggested. There was negligible ergonomic risk for exoscopic surgery and low risk for endoscopic and microscopic surgery. RULA scores are a comprehensive ergonomic assessment. Any one change is unlikely to singularly change ergonomic risk. Rather, the assessment points toward the importance of multifactorial intervention to improve intra‐operative ergonomics. This may also explain why mastoidectomies were associated with a decreased likelihood of MSD despite previous studies suggesting the opposite^3^; this finding may also be due to the wider surgical field mastoidectomies have relative to middle ear surgeries and the RULA score's inability to account for drill vibrations. Thus, while exoscopic surgery may have some ergonomic benefit relative to microscopic surgery, the adoption of this new technology should be pursued in combination with other intraoperative ergonomic adjustments to produce significant and meaningful surgeon benefits.

Our study has limitations. We did not use motion sensors to measure range of motion, or surface electromyography to assess the degree of muscle activation. This would have produced more objective data than manual angle measurements. Our sample size is also relatively small and limited. It features physicians from 2 institutions whose surgical techniques and skills may not fully represent the full range found within otology. In particular, all exoscopic surgeries were performed at Yamagata University, which may limit the external validity of our findings as our sample did not purely consist of surgeons from a single institution performing all 3 surgical modalities. Further studies involving the assessment of all 3 surgical modalities in this manner will be necessary to validate our findings. Our findings may also be subject to the Hawthorne effect, as surgeons may have improved their posture knowing their ergonomics were being assessed. The RULA tool is also an imperfect measure, as it does not assess surgeons' report of MSD risk or bodily characteristics such as body mass index, height, and gender that may influence intraoperative ergonomics. It also does not account for vibrations from the use of an otologic drill. Furthermore, RULA only assesses individual snapshots in time and cannot assess how long certain positions are held for, although our regularly taken intraoperative photos help mediate the impact of this limitation. However, the RULA tool is a validated measure and as such should accurately assess ergonomic risk. Future directions include collecting physician‐reported measures of physical strain and comfort in connection with their intraoperative ergonomic positioning, incorporating more objective measures of body position into our ergonomic assessment, and assessing the ergonomics of other otolaryngologic procedures, surgeries, and surgical modalities to continue improving physician comfort and long‐term musculoskeletal disease risk.

## Conclusion

All 3 modalities for performing middle ear surgery feature low ergonomic risk with exoscopic middle ear surgery demonstrating the lowest risk profile as compared to microscopic and endoscopic ear surgery. Further study should assess how better to optimize ergonomic risk factors to promote surgeon health and longevity.

## Author Contributions

**Matthew E. Lin**, conception and design of work, data acquisition and analysis, interpretation of data, drafting of manuscript, critical revision; **Sheng Zhou**, conception and design of work, data acquisition, interpretation of data, drafting of manuscript, critical revision; **Seiji Kakehata**, conception and design of work, interpretation of data, critical revision; **Tsukasa Ito**, interpretation of data, critical revision; **Seiji B. Shibata**, conception and design of work, interpretation of data, critical revision.

## Disclosures

### Competing interests

None.

### Funding source

None.
